# Staff perspectives on the usability of electronic patient records for planning and delivering dementia care in nursing homes: a multiple case study

**DOI:** 10.1186/s12911-020-01160-8

**Published:** 2020-07-13

**Authors:** Kate Shiells, Angie Alejandra Diaz Baquero, Olga Štěpánková, Iva Holmerová

**Affiliations:** 1 Centre of Expertise in Longevity and Long-Term Care, Faculty of Humanities, Charles University, Prague, Czech Republic; 2 Institute of Biomedical Research of Salamanca (IBSAL), University of Salamanca, Salamanca, Spain; 3 Department of Research & Development, Iberian Research Psycho-sciences Institute, INTRAS Foundation, Zamora, Spain; 4 Department of Cybernetics, Faculty of Electrical Engineering, Czech Technical University in Prague, Prague, Czech Republic

**Keywords:** Assessment, Dementia, Care plan, Electronic health records, Electronic patient records, Nursing home

## Abstract

**Background:**

The electronic patient record (EPR) has been introduced into nursing homes in order to facilitate documentation practices such as assessment and care planning, which play an integral role in the provision of dementia care. However, little is known about how the EPR facilitates or hinders these practices from the end-user’s perspective. Therefore, the objective of this qualitative study was to explore the usability issues associated with the EPR for assessment and care planning for people with dementia in nursing homes from a staff perspective.

**Methods:**

An exploratory, qualitative research design with a multiple case study approach was used. Contextual Inquiry was carried out with a variety of staff members (*n* = 21) who used the EPR in three nursing homes situated in Belgium, Czech Republic and Spain. Thematic analysis was used to code interview data, with codes then sorted into a priori components of the Health Information Technology Evaluation Framework: device, software functionality, organisational support. Two additional themes, structure and content, were also added.

**Results:**

Staff provided numerous examples of the ways in which EPR systems are facilitating and hindering assessment and care planning under each component, particularly for people with dementia, who may have more complex needs in comparison to other residents. The way in which EPR systems were not customisable was a common theme across all three homes. A comparison of organisational policies and practices revealed the importance of training, system support, and access, which may be linked with the successful adoption of the EPR system in nursing homes.

**Conclusions:**

EPR systems introduced into the nursing home environment should be customisable and reflect best practice guidelines for dementia care, which may lead to improved outcomes and quality of life for people with dementia living in nursing homes. All levels of nursing home staff should be consulted during the development, implementation and evaluation of EPR systems as part of an iterative, user-centred design process.

## Background

Nursing homes currently face a multitude of pressures, such as difficulties in recruiting staff, high employee turnover and low staff morale [1]. Added to these pressures is a growing demand for documentation, which has come about from ‘increasing regulatory scrutiny’ [2]. Two of the principal nursing processes which are required to be documented and regularly updated are assessment and care planning. Assessment involves ‘the gathering of data relating to a person’s physical, psychological, and social status’ [3] and may take place in a direct or proxy manner, where information is gathered from family members or by observing individuals. Assessment is often a time-consuming process for staff and can be a stressful activity for the individual, particularly those with dementia [3]. However, it is an important first step in the nursing process, establishing a ‘baseline against which changes can be measured for clinical purposes’ [4]. Furthermore, assessment provides a core set of information from which to identify personalised interventions that maximise an individual’s functionality, so that quality of life can be maintained [4]. These interventions form part of an individual’s care plan [5].

Care plans have been described as ‘prescriptions for nursing care’ [6] and act as a reference for nurses to facilitate continuity of care [7]. Furthermore, care plans are often used to provide evidence of the quality of care which has been delivered [8], in this way, protecting staff in case of complaints [7]. An essential element of the care plan is that it should be personalised to reflect the individual [9]. In addition to containing information about a person’s physical care needs, care plans should also be developed with an individual’s life history in mind, ensuring that care provided is in line with previous lifestyles and routines, which helps to maintain identity and personhood [10]. Care planning plays an important role in the provision of care for people with dementia [11], specifically in nursing homes where, for example, in the United Kingdom, approximately 70% of residents will have a diagnosis of dementia [12].

Defined as an application incorporating ‘the clinical data repository, clinical decision support, controlled medical vocabulary, order entry, computerized provider order entry, pharmacy, and clinical documentation applications’ [13], the electronic patient record (EPR), was introduced to assist with documentation processes such as assessment and care planning. For nursing homes, the EPR has the potential to reduce administrative burdens [14], improve the quality of documentation [15], as well as allow for the identification of care needs [15] and management of long-term conditions more effectively [16]. If EPR systems are interoperable, data can also be shared across healthcare providers [17]. With demands for documentation alleviated, staff potentially have more time to spend with residents providing direct care [18]. The EPR may be particularly valuable for providing care for people with dementia, as it may allow access to detailed background information at the point of care when, for instance, staff may require more information about the cause of an individual’s behaviour [19].

Despite the potential benefits associated with this technology, the EPR has been described as a burden by nursing home staff, which has been linked with issues associated with its usability [20, 21]. In this study, the ISO definition of usability as ‘the extent to which a product can be used by specified users to achieve specified goals with effectiveness, efficiency and satisfaction in a specified context of use’ [22] is adopted. General usability issues associated with the EPR in nursing homes commonly identified in the literature include: separate software programmes for various nursing documentation, which is inefficient [23]; use of incorrect nursing terminology in drop-down menus or templates [8, 24]; lack of space for free data entry [23, 25]; missing forms, meaning paper forms continue to be used [25, 26]; slow log-in processes [27]; and lack of interoperability [23].

The persistent usability issues associated with the EPR in nursing homes highlight the need for a participatory design and evaluation process, where end users’ feedback is gathered as part of an iterative cycle and systems are tailored to their needs [28, 29]. However, previous studies investigating the EPR specifically for care planning in nursing homes have used audit methods to examine the quality and completeness of electronic care plans [8, 30–32], with no input from end users. Problems of usability have also been linked with a lack of consideration of the context in which the EPR is implemented, which has resulted in ‘clashes between the model of health care work inscribed in these tools with the actual nature of work’ [33]. Therefore, through a qualitative lens, this study aims to address the following question: what are the usability issues associated with the EPR for assessment and care planning for people with dementia in nursing homes?

## Methods

### Study design

The study is underpinned by the socio-technical systems theory, which has been suggested as an appropriate framework with which to evaluate Health Information Technology (HIT) such as the EPR [33–35]. This paradigm states that ‘organisational and human (socio) factors and information technology system factors (technical) are inter-related parts of one system, each shaping the other’ [36]. In order to explore these factors, a case study design was selected, which enables the researcher to examine a phenomenon within its natural setting [37, 38]. Furthermore, a multiple case study design was used, which allows for an exploration of the differences and similarities across case studies [36].

### Data collection method

The contextual inquiry (CI) method was used as a means to explore usability issues associated with the EPR in the nursing home setting. CI involves asking users to perform relevant tasks whilst the researcher simultaneously ‘asks questions about what is happening and why’ and ‘how tasks could be improved’, with observations also enabling the researcher to understand contextual issues [39]. Previous research has found the contextual inquiry to be an appropriate method for evaluating the usability of an EPR because, as well as observing an ‘end-user’s interaction’, the researcher is able to develop an ‘understanding of clinical practices’ [40].

For the purposes of this study, participants were asked to show the researcher how they would enter assessment data and create a care plan for an individual with dementia whilst using the ‘think aloud’ method, which allows for a ‘running commentary of [the participant’s] thought process’ [29]. This was followed by a semi-structured interview with open-ended questions, which also provided an opportunity to elaborate on any areas of interest raised during the task. The interview guide can be found in the supplementary materials.

Data collection was carried out in Belgium, Spain, and Czech Republic in order to compare and contrast systems across Europe, and produce common guidelines for EPR development in nursing homes [41]. In Belgium, interviews were carried out in French or English by the first author (KS). In Spain, interviews were carried out by a co-author (ADB) who is a native speaker with the first author present. In the Czech Republic, interviews were conducted either in English by the first author, or in Czech with the assistance of an interpreter who had prior knowledge of the study.

The task and the subsequent interview took place in the office or the room where the device was situated and recorded using a digital voice recorder. The task and interview lasted approximately 60–90 min. The first author (KS) took notes during the task, observing elements such as the environment, the participant’s body language whilst undertaking the task, as well as the device and the EPR software itself.

### Interview guide

The interview guide was designed according to the components of the ‘structural quality concept’ of the Health Information Technology Framework (HITREF) [42]. The HITREF is underpinned by the socio-technical paradigm [43] and was developed in response to a lack of consistent approaches to evaluating HIT, with previous frameworks commonly omitting contextual evaluation [44]. The HITREF has previously been used to map themes relating to the barriers and facilitators to use of an EHR in hospitals, using data from interviews with nurses [45].

The components explored here included: device, software functionality, and organisational support. Two further components, ‘structure and content’, were added by the authors in order to elicit opinions on the language and structure of the EPR forms. Under each component, specific questions were developed from evidence collated from the authors’ prior research [21] and designed to elicit responses about the usability of the EPR for the task.

### Setting

Data collection took place in three nursing homes in Belgium, Czech Republic, and Spain between March 2018 and January 2019. In order to be eligible for this study, the nursing home had to have been using an EPR system for at least 6 months and provide care to people with dementia. Basic characteristics of the nursing homes are provided in Table 1.

**Table 1:** Basic characteristics of nursing homes participating in the study

Date of interviews	Country	Region	Type of nursing home	Total number of beds	Time using the current EPR
March 2018	Belgium	Flanders	Public	316	8 years
June 2018	Spain	Castilla y León	Private	150	8 years
January 2019	Czech Republic	Prague	Public	260	9 months

In Belgium, the EPR system was introduced in 2010. The occupational therapist completes the initial assessment template on the EPR system, using a desktop computer as well as a separate document on paper created by the nursing home more suitable for their needs. This is then scanned and uploaded to the EPR as an attachment. Nurses complete the care plan using a template in the EPR. Nurses use either a desktop computer or a laptop, which contain the full EPR. The auxiliary nurses use a tablet they carry with them, which contains a more simplified version of the care plan.

In Spain, they had been using the EPR system since 2010, however auxiliary nurses do not have access to the system and fill out documentation in notebooks. Currently, when a resident moves into the nursing home, all trained staff have 1 month to fill out their own version of a ‘Programa de Atención Individualizado’ (PAI) on paper, which is a needs assessment and an individualised plan of action according to their field. The PAI is not incorporated into the EPR, and staff add information from this document into various sections of the EPR. Staff who have access to the EPR all use a desktop device.

In the Czech Republic, they had transitioned to a new EPR programme in March 2018, as the previous software was unsuitable for the nursing home environment. Staff are now able to complete the assessments and care plans using the EPR. Due to data protection laws, the nursing home is split into two fields: ‘health’ and ‘social care’ and a dual approach to assessment and care planning takes place. There is also an art therapist working in the home, who assess residents’ needs and plans care in the social domain (hobbies and recreation). Staff members can only view documents in the field in which they work. They mostly use a desktop computer but had introduced tablets for auxiliary nurses 6 weeks prior to the interviews.

### Participants and recruitment

According to research carried out by Nielsen and Landauer [46], carrying out usability testing with 8–10 participants should identify 80% of usability problems, which was the goal sample size. Eight participants were recruited in Czech Republic (female *n* = 8), but only seven in Spain (female *n* = 5; male *n* = 2) and six in Belgium (female *n* = 6). In usability testing, there is also a need to involve a range of users [47] and maximum variation sampling as characterised by job role was used. Table 2 shows the range of different participants according to their role.

**Table 2:** Study participants according to role

Belgium	Spain	Czech Republic
Occupational therapist	Occupational therapist	Social care supervisor
Nurse supervisor	Physiotherapist	Nurse supervisor (*n* = 3)
Nurse	Nurse supervisor (*n* = 2)	Auxiliary nurse supervisor
Auxiliary nurse (*n* = 3)	Nurse	Care quality manager
	Social worker	Social worker
	Home manager	Art therapist

The following inclusion/exclusion criteria for participation was applied:

Inclusion criteria

- Permanent staff member who manages or provides care to residents with dementia.
- Is involved in assessment and care planning.
- Has worked in the nursing home for at least 6 months.
- Has been trained in how to use the electronic documentation system.
- Has been using the electronic documentation system for at least 6 months in order to have had time to familiarise themselves with the system.

Exclusion criteria

- Temporary staff member.

In each of the homes, management were asked to suggest staff who met the inclusion criteria to participate. These staff were provided with an information sheet and consent form. A brief background questionnaire was first given to consenting participants, which was designed to provide an insight into their performance from a historical perspective [29]. For instance, number of years in their role, number of years using the EPR, and self-rated expertise with Information Technnology, ranging from 1 (none) to 5 (excellent). Sample characteristics expressed as means are provided in Table 3.

**Table 3:** Basic sample characteristics expressed as means

Country	Number of participants	Years of experience in nursing home	Length of time using the current EPR system	Self-rated expertise with Information Technology (1–5)
Belgium	6	12.8	5 years	3.4
Spain	7	4.9	4.5 years	4.1
Czech Republic	8	2	8.3 months	3.6

### Data analysis

Interviews from Belgium were transcribed by the first author (KS). Interviews from Spain and Czech Republic were transcribed by a professional transcription company then translated into English by two authors (KS, ADB). Theoretical thematic data analysis was carried out, which allows data to be coded for a specific research question and according to a theoretical pre-conception [48]; in this instance, socio-technical systems theory. Data was coded into sub-themes according to each of the a priori, overarching components from the Structural Quality evaluation concept of the HITREF Framework [42]. The first author (KS) carried out thematic analysis until no new sub-themes emerged and saturation was reached. Transcripts were then checked by a co-author (ADB) for any additional sub-themes. KS made the final decision. Data analysis was carried out using ATLAS.ti software.

## Results

The overarching components from the adapted HITREF framework and corresponding sub-themes are described below. Table 4 summarises results by component.

**Table 4:** Summary of themes arising from the data

HITREF Component	Sub-theme	Considerations
**Device**	Type of device	Portable device allows staff to access care plans and record assessments at point of care.
		Desktop device may be preferable for writing longer documents.
		Portable device should be discrete and have a mute function.
	Number of devices	Staff involved in assessment, care planning, and care provision should ideally have access to their own device so that important information can be immediately accessed or recorded before and after care delivery.
**Software functionality**	Drop-down menus	Incorporation of drop-down menus that can be customised by the nursing home may save time during assessment and care planning and act as prompts.
		Space for free data entry may still be required.
	Customisable terminology	Nursing home should be able to customise terminology used in assessment and care plan templates that is appropriate for staff level.
	Alerts about changes in a resident’s condition	EPR should alert staff on entering the programme to any significant changes in a resident’s condition, such as admission to hospital.
	Alerts to update care plans	EPR should alert staff when care plan requires updating.
		Nursing home should be able to choose whether old care plan is automatically deleted or not.
	Interoperability	Interoperable system would mean less time is spent entering information from hospital discharge forms into the EPR and clarifying unclear information.
		Interoperable system would allow nursing home staff to remain up to date about a resident’s condition whilst in hospital and plan for their return.
**Structure & content**	Assessments for dementia	EPR should include assessment scales relevant to dementia care, for instance: -QUALID Scale -Mini-mental State Examination -Barthel Index
		EPR may need to incorporate assessment templates based on observations of individuals for those at advanced stages of dementia.
		Various assessment templates should be incorporated for all relevant staff, including assessment of social needs.
		Assessments should be customisable for client group and not force staff to enter irrelevant information.
	Care planning for dementia	EPR should alert staff to any specific changes in physical health that could impact on a resident’s behaviour, such as: -Changes in eating or drinking habits. -Changes in bowel habits. -Changes in body temperature.
		Care plan template should be customisable, but may need to prompt staff to include the following: -Type of dementia. -Key contact person such as family member or friend. -Life history of resident. -Information about routines. -Information about hobbies, -Past profession and whether this was enjoyed by the individual or not.
	Improvements in structure	Staff require a table in the EPR where they can record all observations (e.g. blood pressure, temperature, oxygen saturation, heart rate) in the same place.
		EPR should allow staff to easily access assessments and care plans of other staff, for instance, by incorporating tabs according to each profession (e.g. physiotherapist, occupational therapist) that appear on the screen.
		EPR may need to include space to record how long each task of the care plan takes to complete.
**Organisational support**	Access	EPR may need to offer customisable access to assessments and care plans for staff according to their level of training. For instance, management may decide access to dementia diagnosis should be restricted.
	Training	Training ‘on the job’ should be offered as an alternative to classroom-based teaching.
		Training should be customised according to previous experience with technology.
		Ongoing training should be offered at appropriate times.
	System support	Ongoing communication with EPR developers is crucial for appropriate updates to be made to the programme.
		A named person within the nursing home who maintains contact with the developer and to whom problems can be addressed may be appropriate.
		Onsite support for immediate IT issues may also be important.

### Device

#### Type of device

A tablet device was preferred by many participants as it could be transported in the nurses’ trolley for easy access to care plans:

*When you are with a resident who needs care, I do not have to go downstairs to see the treatment.* (Nurse).

In two homes, staff are currently carrying out the initial assessment with residents in their rooms on paper, then entering information into the EPR. Several staff members said it would be faster if they had a tablet device to record the assessment. However, when writing care plans, nurses preferred to use a desktop computer as they found the keyboard easier to use for long documents. Staff were also concerned about tablets becoming broken or lost.

Staff had mixed opinions on whether devices should be used in residents’ rooms. In one home, this was no longer the case as staff felt it made their rooms ‘like a convenience store’ (Care quality manager). In the home where they were using devices in rooms, the noise of the device made one participant uncomfortable:

*It’s horrible. There is a human being, and you come and beep, beep like a robot. What is this, science fiction?* (Auxiliary nurse).

#### Number of devices

Several staff complained about having to share devices and wait until they became available. Typically, staff need to use the device at the same time:

*It’s terrible during lunchbreak because the clients are eating and sleeping so everybody is on the computer.* (Art therapist).

There were concerns that by sharing devices, staff were prevented from viewing updated care plans before delivering care. One participant highlighted how often she did not know someone was in hospital until she visited their room and found that they were not there.

### Software functionality

#### Drop-down menus

Participants across all three homes noted that it takes time to type free text into the EPR. As a result, despite having a portable device for data entry at the point of care, staff often carried out administrative work after delivering personal care to all residents, in order to prioritise time spent with individuals. This is even more crucial when caring for people with dementia:

*The tablet is extra work, and for people with dementia, it’s very important for me to give them extra time.* (Auxiliary nurse).

It was suggested that users would benefit from writing less if the software incorporated drop-down menus. In particular, nurses found a body chart with drop-down menus to record wound care intuitive:

*You don’t have to think what you have to do. You can select “clean at 7am with Betadine”.* (Nurse).

However, staff noted that drop-down menus should be customisable, and that space for free text may still be required.

#### Customisable terminology

There were complaints that the terminology used in some EPRs was complex for staff with less training in the field:

*There are a lot of terms, which often a basic caregiver doesn’t understand.* (Care Quality Manager).

However, in the Czech home where they had recently introduced a new programme, there was a functionality that addressed this issue:

*It has an advantage, that you can adjust the phrases as you please so that everyone can understand.* (Care Quality Manager).

#### Alerts about changes in a resident’s condition

Staff in all homes currently use an internal messaging application within the EPR or hold regular meetings to communicate changes in a resident’s condition and how to adjust care. However, there is no functionality to alert staff based on data entered into the EPR:

*The program does not alert us at all. We have a multidisciplinary meeting every day.* (Nurse supervisor).

In order to increase awareness of a resident’s condition, staff would like an alert system:

*If there will be any alarm when I open [the EPR] and it tells me the most important stuff it will be brilliant […] if it shows that this person died or this person fell down.* (Art therapist).

#### Alerts to update care plans

In the Czech home, staff are warned that the care plan needs updating when a red circle appears next to a resident’s name. In the two other homes, the EPR does not provide alerts and staff keep a record on paper. Staff in the Czech home are required to evaluate the care plan every 3 months. At this time, the previous care plan is automatically deleted and they should rewrite it, which causes frustration. However, not everyone agreed that this was a negative functionality of the EPR:

*I personally don’t mind, because at least the staff are forced to think about the current care plan.* (Nurse).

In Belgium, the auxiliary nurses are required to tick off each section of the care plan on the tablet which has been completed, but this can sometimes be forgotten during a busy shift, leading to repercussions:

*What happens is if inspection comes, they will say “you have not washed this woman today”, you say “no I forgot [to enter it on the care plan]”, but on the computer it shows that she hasn’t been washed.* (Auxiliary nurse).

In the home in Belgium where they have two types of devices, there is no alert to remind staff to sync the updated care plan with the tablets so that auxiliary nurses provide correct care:

*Sometimes people change the care plan […] and they don’t always change it here in the tablet.* (Auxiliary nurse).

#### Interoperability

In all three homes, the EPR was not interoperable. When residents move to the home, they bring a paper report with details of medical history, which has to be manually entered into the EPR. Nurses find this frustrating and they often need to call the hospital to clarify unclear information:

*We get no information from hospitals, only on paper, the old-fashioned way.* (Nurse).

### Structure & content

#### Assessments for dementia

Often core assessment scales were missing from the EPR, which is frustrating for staff as they need to complete these scales on paper. Scales that staff said they require for assessing people with dementia were the Quality of Life in Late-Stage Dementia Scale (QUALID) [49], the Mini Mental State Examination (MMSE) [50], and the Barthel Index [51, 52]. Furthermore, it was highlighted how staff also require access to assessment templates based on observations, which may be more appropriate for assessing people with advanced dementia with communication problems or those who become anxious during a typical assessment:

*A lot of people like it that you come and speak about the past and the future, and just have a talk. Others are scared and think that you are asking questions about something bad.* (Occupational therapist).

In the newly introduced system in the Czech home, many of the areas in the electronic assessment forms were said to be inappropriate for the client group, where questions were aimed at the assessment of patients with mental health problems. Whereas for people with dementia:

*there are no options that we might like to have clicked, that the clients are, for example, chronically or acutely confused.* (Nurse).

Staff in the Spanish home were also frustrated that they could not add assessment templates themselves:

*The program does not offer flexibility, they give you the formats, they are the ones that exist and you cannot adapt them. The program is standardised for all nursing homes, but each nursing home has its own characteristics.* (Home manager).

#### Care planning for dementia

Care planning plays an important role in the work of nurses, and the EPR should facilitate this process. However, in the Czech nursing home, the old EPR had been replaced, partly because it was inappropriate for nursing staff to develop their care plans:

*‘It’s a programme for doctors [ … ] it’s not suitable for nurses [ … ]. There was no space for a care plan, just to add medical history and a few assessment tests.’* [Care quality manager].

When asked about the most important information in a care plan that staff needed to know about a resident with dementia, a common answer was the need to be alerted to any deterioration in physical health. For example, changes in eating, drinking or bowel habits, and changes in temperature, all of which could indicate possible infection and explain recent behavioural changes. It was emphasised that such information is particularly valuable for those residents with difficulties communicating verbally that they are unwell. Furthermore, staff require contact information of a family member or friend who may have greater insight into reasons linked to any changes in a resident’s behaviour. Information about the type of dementia is also needed:

*You have several sorts of dementia and sometimes you have dementia without forgetting things, so the kind of dementia is important.* (Occupational therapist).

In addition to information about physical health, a number of staff highlighted the importance of obtaining a life history in order to obtain a holistic picture of an individual:

*I want to put the stories in [the EPR] to remind people that this person who is lying on the bed was really a hero in his life.* (Art therapist).

In order to create the most natural environment for the resident, knowledge about hobbies, past routines, and professions are important:

*What routines did this person have before coming to the home, for example, if he loves to walk in the park, if he needs a coffee at two o clock in the afternoon, so I don’t interrupt his routines.* (Art therapist).

It was emphasised that creating a care plan template for people with dementia was not always possible, and staff would prefer to personalise care plans on the EPR:

*For every person, dementia is different. I have to make my own plan [… ] the development of the disease is also different.* (Art therapist).

#### Improvements in structure

There were examples across all three homes where the amount of information staff were required to fill in was more than necessary, and other instances where there was not enough space to record what was needed:

*This one for falls, it does not reflect everything that our [paper] fall sheet reflects*. (Supervisor).

Specific improvements in structure included a table where all observations can be entered and viewed together. Trained staff also need to be able to access information recorded by each staff member. In one EPR there are tabs for each profession where they can easily access each professional’s assessment and care plan, which is important, as explained by one participant:

*Families always come to ask the nurse. They ask you about the physio, the therapist, the doctor and everyone. You have to know everything.* (Nurse).

One auxiliary nurse wanted to be able to enter next to each step of the care plan how long it takes them:

*This system does not show how much time you put in to caring for each person. It can be that you take more time with someone because they are slower, or they don’t understand.* (Auxiliary nurse).

### Organisational support

#### Access

Access to the EPR differed across the homes. In Spain, auxiliary nurses could not access the EPR and were required to write notes by hand, and in Belgium where auxiliary nurses had basic access to care plans via the tablet, they were frustrated with the limited amount of information they could access:

*[The tablet] shows what you have to do, but not how the person is. So, it doesn’t show if the person has behavioural issues.* (Auxiliary nurse).

Some trained staff felt that due to the complexity of the system, access should be restricted so that documentation is not accidentally deleted. One participant believed auxiliary nurses should not have access to dementia diagnosis, as they may treat the individual differently:

*We have always tried not to work with that person on the basis of his diagnosis, but on the basis of the personality.* (Nurse).

Others believed auxiliary nurses should have access to the full EPR, including dementia diagnosis, in order to provide the most person-centered care.

#### Training

When asked about training, the majority of participants said that learning ‘on the job’ was more useful than attending a course, as they found the EPR intuitive:

*In the beginning it was just pure information [ … ] but I’m the type, I just need to see it.* (Social Worker).

This may be linked with age and prior experience with technology:

*I basically grew up with these kinds of technology. It really didn’t cause me a problem.* (Nurse).

One participant with limited experience of technology would have liked more basic training from colleagues in the home, and the option of booking extra training when required. Another nurse felt overwhelmed when starting her role, and would have liked more time to learn to use the EPR:

*When you start as a new staff member, then there’s a lot you have to learn, and you have to learn it very quickly. There’s no time to practice.* (Nurse).

#### System support

The importance of contact with developers on an ongoing basis was highlighted. In the Czech home, staff could write notes and feedback any problems directly to the developer, who also had remote access and could look into any issues quickly. However, this was not the case in the Spanish home, and staff complained about a lack of updates:

*We use equipment that is not sufficiently agile.* (Supervisor).

In all homes, there were allocated staff who were in charge of reporting issues to the developer, a system which worked well:

*I tell my boss if there is a problem with the EPR because I am not the relevant person who can call. There is a structure. It would be a mess if anyone can call.* (Art therapist).

Finally, in the home in Belgium, there was a specific onsite employee who was responsible for managing IT systems in the home, whom staff could approach in order to resolve any immediate issues with the EPR.

## Discussion

The observations and interviews carried out across the three nursing homes have contributed towards a greater understanding of the ways in which certain technical elements of the EPR are linked with the usability of the system for assessment and care planning, particularly for people with dementia. They also allowed for an insight into organisational aspects of the nursing homes, and the ways in which these may be facilitating or hindering the adoption of the EPR.

A common issue associated with the EPR systems across all three homes was the way in which they were not customisable. Participants spoke about how they wished to adjust various elements of the EPR to meet the specific needs of the nursing home and staff, such as work practices, and the needs of the individuals who live there. This highlights how a close relationship between the developer and the end user as part of a user-centred design (UCD) process is important [53].

In regards to devices, portable devices accessible at the point of care were often preferable. However, some nursing staff said they preferred working on a desktop device due to ease of use. This stresses the need for all levels of nursing home staff to be consulted and individual requirements according to role and experience with technology to be taken into account during system design [54]. There were also concerns amongst several staff that the use of technology in the proximity of residents was intrusive and had led to a reduction in the personal aspect of delivering care, which is in line with previous research showing that HIT may be dehumanising care [55]. The need for unobtrusive devices is of particular importance when taken in the context of dementia-friendly nursing homes, one principle of which states that personalised environments encouraging ownership are crucial [56].

Developers should ensure that software facilitates the assessment and care planning process, for instance, through customisable drop-down menus, which may reduce time spent on entering information. A number of participants also described the benefit of a system that provides alerts in a resident’s condition and directs them to the appropriate care, which could be achieved through the incorporation of a clinical decision support system (CDSS). CDSS has been defined as a system providing ‘evidence-based recommendations, alerts, or reminders using patient-specific information to improve clinical reasoning and decision making’ [57]. In the nursing domain, electronic nursing care reminders (NCRs), a type of CDSS incorporated into the EPR in the form of pop-up alerts with details of care, were found to be associated with decreased reports of missed nursing care [57]. However, Mitchell and Ploem [58] highlight the ‘tension’ between the potential benefits and risks of more advanced versions of CDSS that use machine learning and are based on data from real patients. Risks include errors of analysis and a loss of trust in healthcare providers, as well as failure to secure data confidentiality [58].

A lack of interoperability was described by staff in all three nursing homes, which is a common shortcoming of EPR systems [59]. A review of the literature on the management of dementia in primary care found that in order for the effective coordination of dementia care to take place, it is critical for information to be shared across healthcare providers [60]. Access and sharing of care plans across those services previously supporting an individual in the community through the means of an interoperable EPR system would allow continuity of care as the individual moves into the nursing home [61, 62]. However, interoperability is also reliant upon the consistent use of terminology across EPR systems, as well as common standards in data quality and a common architectural model [63]. Therefore, customisable EPRs are unlikely to be compatible with interoperable systems and may in part explain why interoperable EPRs are ‘yet to become a reality’ [64].

Consideration of the nursing home population during the design process is also necessary. This was evident in one of the nursing homes, where the EPR was designed for patients of mental health services and inappropriate for planning dementia care. Moreover, in one home, there was no specific place to record dementia diagnosis. Staff also reported that they require a large and varied amount of information in order to plan and deliver care for an individual with dementia. Prior research has shown that staff access to a life history of an individual with dementia is linked with increased understanding and empathy towards individuals displaying neuropsychiatric symptoms of dementia [65]. Furthermore, due to the range of dementias and their different associated needs, which will also vary according to each individual, staff need space to create personalised care plans with individualised goals, in addition to entering standard information required by local and national best practice guidelines for care planning [62].

Although this study has primarily focused on the ways in which technical components of the EPR were impacting usability for dementia care, a comparison of organisational policies and practices across the three homes also revealed the importance of certain factors implicit in the successful adoption of an EPR system [33]. Evidence from a number of studies has shown that training is key if effective implementation of EPR is to take place in nursing homes [30, 66]. In this study, training ‘on the job’ was more widely-preferred over classroom-based teaching and should be tailored to the individual’s level of experience with IT. Secondly, system support, which may take the form of a specific individual onsite was specified as crucial. This is in line with prior research, which found that onsite support was one of five key elements associated with the successful implementation of EPR in nursing homes [67, 68].

The question of who should have access to the EPR was also raised, particularly in regards to auxiliary nurses, who had no access to the EPR in the Spanish nursing home, and reduced access in the nursing home in Belgium. This was linked with fears held by management or nursing staff that auxiliary staff may not be able to use the system correctly, or that they may treat residents differently if they had access to clinical information, in particular, their dementia diagnosis. Whilst there is previous research to suggest that using EPR reduced the amount of time auxiliary nurses spent with residents, it was also found to have increased their accountability [67].

### Limitations

Recruitment within each nursing homes was challenging due to lack of available staff and time. The goal sample size was 24 participants, although only 21 participants were recruited. However, the authors agreed that saturation had been reached whilst coding transcripts. Furthermore, although this project aimed to compare similar nursing homes across three countries, this was problematic due to the different systems of care across Europe. In particular, the fact that one nursing home was privately funded whereas two were public could have meant results were not comparable. In addition, whilst the EPR had been in use in the nursing homes in Belgium and Spain for 8 years, in the Czech Republic they had recently introduced a new EPR 9 months prior to the time of the interviews. This may have meant that staff had had less time to familiarise themselves with the full functionalities or limitations of the new EPR.

Selection bias may have occurred, as management were asked to select staff for interview, according to their availability. However, staff known to have specific opinions towards the EPR may have been selected. Furthermore, a greater number of managers and supervisors were interviewed in the Czech Republic than frontline workers, which may have also biased responses in favour of the EPR, especially if they had been involved in its design. Finally, translation of transcripts from their original language into English may have caused some nuances to be lost, and as interviews took place in the nursing home often surrounded by other staff, it may have meant some participants were reluctant to discuss negative issues.

### Future research

Future research should consider exploring the usability of the EPR with auxiliary nurses in more detail as they are key staff members often at the frontline in regards to care delivery. In addition, more research into the particular guidelines for dementia assessment and care planning in each of the countries is required to develop country-specific guidelines for EPR systems.

## Conclusions

This qualitative exploration of staff perspectives of EPR in three nursing homes has revealed that the three EPR systems are both helping and hindering staff to plan and deliver care. All homes highlighted the importance of customisable systems, and the lack of specific characteristics needed to effectively plan and deliver care for people with dementia. People with dementia in nursing homes may have more complex needs in comparison to other residents. Therefore, EPR systems introduced into the nursing home environment should reflect best practice guidelines for dementia care, which may lead to improved outcomes and quality of life for people with dementia. Furthermore, all levels of nursing home staff should be consulted during the development, implementation and evaluation of EPR systems as part of an iterative, user-centred design process.

## Supplementary information

**Additional file 1.** Interview Guide

## Data Availability

The datasets used and analysed during the current study are available from the corresponding author on reasonable request.

## Abbreviations

CDSS: Computerised Decision Support System
CI: Contextual Inquiry
EPR: Electronic Patient Record
HIT: Health Information Technology
HITREF: Health Information Technology Evaluation Framework
MMSE: Mini Mental State Examination
NCR: Nursing Care Reminder
QUALID: Quality of Life in Late-Stage Dementia Scale
SNL: Standardised Nursing Language
