# Supplementary material for: Staff perspectives on the usability of electronic patient records for planning and delivering dementia care in nursing homes: a multiple case study
Source: BMC Med Inform Decis Mak. 2020 Jul 13;20:159. doi: 10.1186/s12911-020-01160-8 (PMC7359585; doi:10.1186/s12911-020-01160-8)
Supplement: Supplementary file 1 — Additional file 1. Interview Guide [file 12911_2020_1160_MOESM1_ESM.docx]

Interview Guide

1. **Device**

What is your opinion on the type of device you use?

To what extent do you think the device is suitable for the nursing home environment?

What is your opinion on the amount of devices in the home?

Have the residents or visitors ever expressed an opinion on the device?

1. **Software functionality**

How easy do you find filling out assessments?

How easy is it to create a care plan from assessment data?

How easily can you access resident information from other care providers? E.g. hospitals?

How easily can you create trends from data?

Are you reminded to create or update assessments and care plans?

How do you receive alerts about changes in a resident’s condition?

Which changes to the system would help you to complete assessment and care planning forms more easily? Are there any applications that could help you with assessment and care planning?

Which changes to the system would help you to access assessment forms and care plans more easily?

1. **Structure and Content**

How do you feel about the amount of information you collect about residents for assessment and care planning?

What is your opinion on the language used in the forms? Is standardised nursing terminology used?

In your opinion, to what extent do forms reflect best practice in dementia care?

What is your opinion on the layout of forms? Are there sections missing?

How easily can you access care plans?

How easily can you access information about dementia diagnosis etc.?

What is most important for you to know about the residents with dementia you care for?

1. **Organisational support**

How much training did you receive?

Can you describe the training?

Was it sufficient? Is it ongoing?

If there is a problem with the EPR, how do you access help? Is this sufficient?
